# Lipid Metabolism in Gastrointestinal Malignancies: Exploring Dysregulation, Biomarkers, and Treatment Strategies

**DOI:** 10.1002/cam4.70975

**Published:** 2025-05-20

**Authors:** Yan An, Huihui Song, Hongyan Qiu, Jun Jiang, Junfeng Shi

**Affiliations:** ^1^ Department of Anesthesiology Affiliated Hospital of Shandong Second Medical University Weifang China; ^2^ Obstetrical Medicine Center, Weifang People's Hospital Shandong Second Medical University Weifang China; ^3^ Department of Endocrinology and Metabolism, School of Clinical Medicine, Affiliated Hospital of Shandong Second Medical University Shandong Second Medical University Weifang China; ^4^ Clinical Research Center Affiliated Hospital of Shandong Second Medical University Weifang China

**Keywords:** biomarkers, gastrointestinal malignancies, lipid metabolism, lipid‐targeted therapy, TME

## Abstract

**Background:**

Gastrointestinal malignancies are a major public health concern worldwide, characterized by high incidence and mortality rates. Despite continuous advancements in existing treatment methods, overall survival rates remain low. Lipid metabolism plays a crucial role in the occurrence, progression, and treatment of gastrointestinal malignancies. Its involvement in the metabolic reprogramming of tumor cells, regulation of the tumor microenvironment, and drug response has become a research hotspot.

**Materials & Methods:**

This review summarizes current research related to lipid metabolism mechanisms, biomarkers, and therapies in GI cancers, with emphasis on its interaction with the tumor microenvironment.

**Abbreviations**
ACAT1: acetyl‐CoA acetyltransferase 1
ACC: acetyl‐CoA carboxylase
ACLY: ATP citrate lyase
Ala: alanine
AMPK: adenosine 5′‐monophosphate (AMP)‐activated protein kinase
Arg: arginine
CAFs: cancer‐associated fibroblasts
CD155: cluster of differentiation 155
CD36: cluster of differentiation 36
CD47: cluster of differentiation 47
Cir: circulation
CPT1: carnitine palmitoyltransferase1
CTLA‐4: cytotoxic T‐lymphocyte‐associated protein‐4
Cys: cysteine
ELOVL: long chain fatty acid elongase
ER stress: endoplasmic reticulum stress
FA: fatty acids
FAO: fatty acid oxidation
FAS: fatty acid synthesis
FASN: fatty acid synthase
FFAs: free fatty acids
Gln: glutamine
Glu: glutamate
GSH: glutathione
HIF‐1α: hypoxia‐inducible factor‐1α
HLA‐G: human leukocyte antigen G
HMGCR: 3‐hydroxy‐3‐methylglutaryl‐CoA reductase
Kyn: kynurenine
Lac: lactate
LAG‐3: lymphocyte activation gene‐3
LDLR: low‐density lipoprotein receptor
LPA: lysophosphatidic acid
LPC: lysophosphatidylcholine
mTOR: mammalian target of rapamycin
MUFA: monounsaturated fatty acid
NK cells: natural killer cells
NO: nitric oxide
nSMase2: neutral sphingomyelinase 2
PD‐1: programmed death‐1
PD‐L1: programmed death‐ligand 1
PGE2: prostaglandin E2
PIP3: phosphatidylinositol 3,4,5‐trisphosphate
Pro: proline
S1P: sphingosine‐1‐phosphate
SAFA: saturated fatty acid
SCD 1: stearyl‐coenzyme A‐desaturase 1
SOAT1/ACAT1: sterol O‐acyltransferase1
SREBP‐1: sterol regulatory element‐binding protein 1
SREBP‐2: sterol regulatory element‐binding protein 2
TCA Cycle: tricarboxylic acid cycle
Teffs: effector T cells
TG: triacylglycerol
TIM‐3: T cell immunoglobulin domain and mucin domain‐3
Tregs: regulatory T cells
XBP‐1: X‐box binding protein‐1

## 1 Introduction

Gastrointestinal malignancies (GI cancers), including esophageal, gastric, colon, and rectal cancers, are among the leading causes of cancer‐related deaths worldwide. Despite advancements in traditional treatments such as surgery, chemotherapy, and radiotherapy, the overall survival rate for GI cancers remains low [1, 2, 3]. This is largely attributed to their complex pathogenesis and high recurrence rates [4, 5, 6]. Therefore, exploring novel molecular mechanisms and therapeutic targets is critical for improving the prognosis of patients with GI cancers.

Lipid metabolism plays a central role in the occurrence and progression of GI cancers [7, 8]. Lipids are not only essential components of cell membranes but also play key roles in cell signaling, energy metabolism, and the regulation of the tumor microenvironment [9, 10, 11]. Compared to normal cells, cancer cells undergo metabolic reprogramming to redistribute lipid metabolic pathways, meeting the demands of rapid proliferation and survival [12, 13, 14, 15, 16, 17]. These metabolic abnormalities not only drive malignant tumor progression but also offer potential biomarkers and targets for diagnosis and treatment.

In recent years, studies on lipid metabolism have increasingly highlighted its importance in GI cancers [18, 19]. Abnormal lipid levels and enzyme overactivation (e.g., FASN and ACC) are linked to higher cancer risk and aggressiveness [20, 21, 22, 23, 24]. Furthermore, lipid metabolic dysregulation also promotes cancer cell growth and metastasis by affecting immune regulation and metabolic signaling within the tumor microenvironment (TME) [17, 25].

This review systematically examines the mechanisms of lipid metabolism in GI cancers. Starting with normal lipid metabolic pathways, it focuses on their dysregulation in cancer and summarizes the diagnostic potential of related biomarkers. Additionally, the latest developments in lipid metabolism‐targeted therapeutics are evaluated, discussing their clinical challenges and future directions. By integrating the latest research findings, this review aims to provide new insights into the diagnosis, treatment, and prognosis management of GI cancers. Notably, this review places particular emphasis on gastrointestinal tumors, including gastric, colorectal, and hepatocellular carcinomas, to highlight cancer‐type‐specific metabolic features.

## 2 Normal Lipid Metabolism Pathways

**FIGURE 1:**
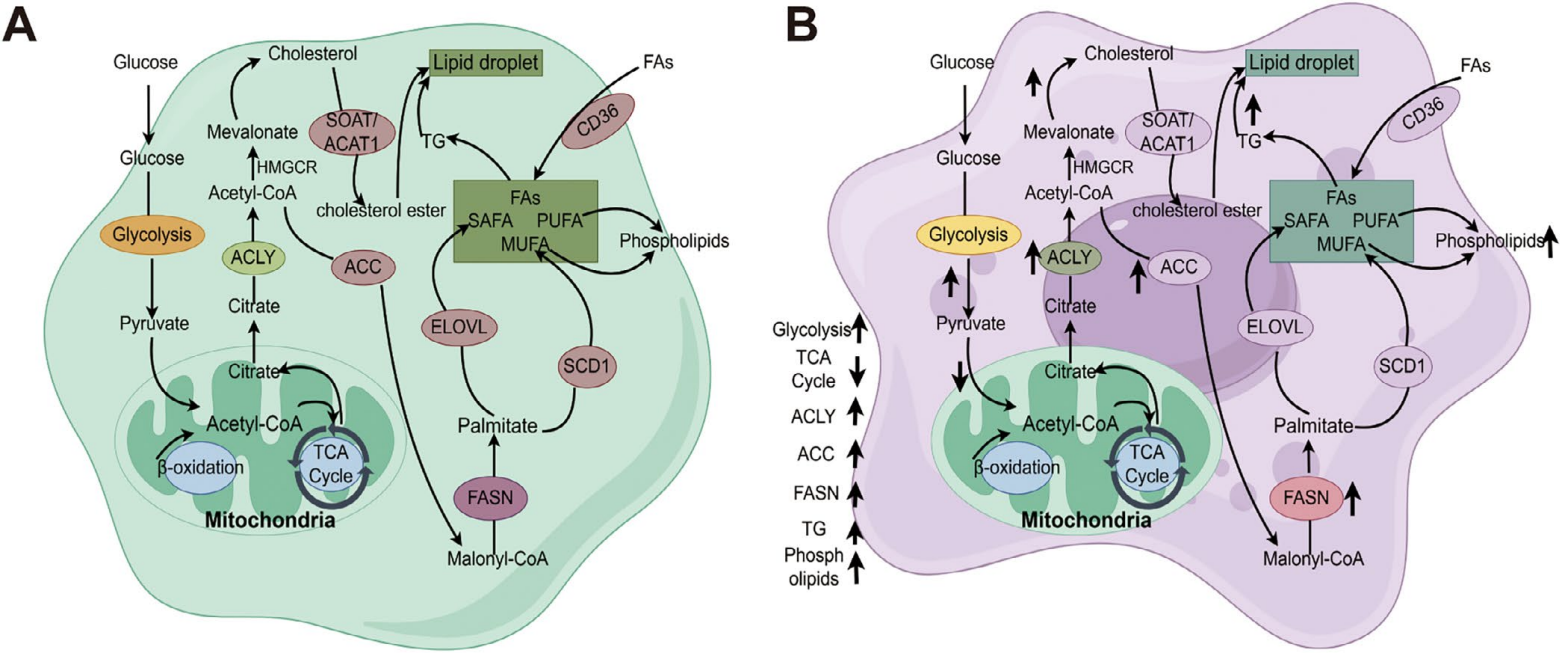
Schematic diagram of lipid metabolic pathways in normal cells (A) and cancer Cells (B). This diagram contrasts the lipid metabolism pathways in normal cells and cancer cells. In cancer cells, processes like lipogenesis, fatty acid oxidation (FAO), and cholesterol synthesis are disrupted. Arrows indicate the direction of regulation: ↑ for upregulation and ↓ for downregulation.

Lipid metabolism is fundamental to cellular homeostasis, involving three interrelated pathways: lipid synthesis, fatty acid oxidation (FAO), and cholesterol metabolism [26, 27]. These pathways dynamically respond to physiological needs such as membrane formation, energy production, and signal transduction [9, 10, 11] (Figure 1A).

### 2.1 Lipid Synthesis

Lipid synthesis begins with acetyl‐CoA, primarily derived from glucose metabolism. Acetyl‐CoA carboxylase (ACC) converts acetyl‐CoA to malonyl‐CoA, followed by fatty acid synthase (FASN) catalyzing the formation of palmitate. These products serve as precursors for membrane phospholipids and energy storage lipids. The process is tightly regulated by sterol regulatory element‐binding proteins (SREBPs), aligning lipid production with cellular demands.

### 2.2 Fatty Acid Oxidation (FAO)

FAO is a key energy‐producing process, particularly during nutrient deprivation. Long‐chain fatty acids are transported into mitochondria via the carnitine system, where β‐oxidation produces acetyl‐CoA that fuels the tricarboxylic acid (TCA) cycle and oxidative phosphorylation. AMP‐activated protein kinase (AMPK) activates FAO by promoting CPT1 and inhibiting ACC under low‐energy conditions.

### 2.3 Cholesterol Metabolism

Cholesterol, essential for membrane integrity and hormone synthesis, is regulated via de novo synthesis, dietary uptake, and esterification for storage. Sterol regulatory element‐binding protein 2 (SREBP‐2) governs this process by modulating key enzymes such as HMG‐CoA reductase (HMGCR) and low‐density lipoprotein receptor (LDLR).

### 2.4 Integration of Lipid Metabolic Pathways

These lipid metabolic processes are intricately coordinated to maintain metabolic flexibility. In energy‐rich states, lipid synthesis is upregulated, while in energy‐deficient conditions, FAO predominates. Cholesterol metabolism integrates into this balance by maintaining membrane composition and cellular signaling [28, 29, 30, 31, 32].

However, in cancer, this homeostasis is disrupted. Tumor cells undergo lipid metabolic reprogramming to support rapid growth, with enhanced lipid synthesis for membrane biogenesis, increased FAO for energy, and altered cholesterol metabolism facilitating signal transduction and invasion [33, 34, 35, 36, 37].

## 3 Altered Lipid Metabolism in Cancer

During cancer progression, alterations in lipid metabolism are considered a hallmark of tumor initiation and development [38, 39]. Compared to normal cells, cancer cells exhibit significant reprogramming of lipid metabolism to meet the demands of rapid proliferation and adapt to the harsh tumor microenvironment [40, 41] (Figure 1B). Moreover, the genetic background of cancer cells, including molecular subtypes, can influence the extent and nature of these metabolic alterations, further highlighting the importance of integrating genetic and metabolic perspectives in understanding cancer progression [42].

### 3.1 Reprogramming of Lipid Synthesis and Uptake

Cancer cells often enhance lipid supply by activating de novo lipogenesis and fatty acid uptake pathways to meet their biosynthetic and energetic needs [43, 44, 45, 46, 47, 48, 49]. The key enzymes in de novo lipogenesis, fatty acid synthase (FASN) and acetyl‐CoA carboxylase (ACC), are frequently upregulated in gastrointestinal (GI) cancers [20, 21, 22]. FASN catalyzes the synthesis of palmitate, a major saturated fatty acid for membrane and energy demands, while ACC produces malonyl‐CoA, a precursor for fatty acid elongation [23, 24]. Overexpression of FASN and ACC is associated with increased tumor aggressiveness, poor prognosis, and treatment resistance. These enzymes not only sustain rapid cell proliferation but also participate in oncogenic signaling, making them promising therapeutic targets and prognostic biomarkers in GI malignancies [42].

In addition to enhanced synthesis, the fatty acid uptake mechanisms of cancer cells are also markedly upregulated. Cancer cells increase the expression of fatty acid transport proteins, such as CD36, to efficiently absorb free fatty acids (FFAs) from the tumor microenvironment [50, 51]. This exogenous lipid supply complements their metabolic needs, further supporting their growth and proliferation.

### 3.2 Adaptive Regulation of Fatty Acid Oxidation (FAO)

Fatty acid oxidation (FAO) serves as a crucial energy source for cancer cells under conditions of hypoxia or nutrient deprivation [35]. This metabolic flexibility allows cancer cells to survive and proliferate in hostile environments. Under low oxygen conditions, hypoxia‐inducible factor‐1α (HIF‐1α) is upregulated, significantly enhancing the expression of FAO‐related genes. This leads to increased FAO activity, ensuring that cancer cells can generate ATP through fatty acid oxidation despite limited oxygen availability [52]. The upregulation of HIF‐1α and its downstream effects on FAO are often influenced by the genetic landscape of cancer cells, particularly mutations that stabilize HIF‐1α under normoxic conditions [53].

In nutrient‐deprived conditions, AMP‐activated protein kinase (AMPK) plays a pivotal role in promoting FAO by regulating the activity of acetyl‐CoA carboxylase (ACC). This regulation helps cancer cells adapt to changes in the microenvironment, providing an energy source to support their survival and continued growth [36].

### 3.3 Role of Lipids in Signal Transduction and Membrane Remodeling

The reprogramming of fatty acid metabolism in cancer cells provides not only structural components but also plays a critical role in signal transduction. Fatty acids and phospholipids act as signaling molecules that activate key oncogenic pathways, such as the PI3K/Akt/mTOR pathway, enhancing the survival, migration, and invasion capabilities of cancer cells [54]. In particular, phosphatidylinositol derivatives (e.g., PIP3) are pivotal in regulating cell proliferation and anti‐apoptotic signaling, promoting tumor progression [55]. These lipid‐mediated signaling pathways are often regulated by genetic mutations in cancer cells, which can further drive oncogenic signaling and tumor progression [42].

Furthermore, alterations in lipid metabolism significantly influence the composition and fluidity of cell membranes. Cancer cells adjust the proportion of membrane lipids to increase plasticity and fluidity, facilitating their migration to adjacent tissues [56].

### 3.4 Role of Lipids in Precursors for Other Biologically Active Substances

Lipids are precursors of many biologically active substances, which play a key role in the growth, survival, and metastasis of cancer cells [57]. For example, phospholipids are the main components of cell membranes and also serve as precursors for various signaling molecules. Phospholipids can be hydrolyzed by enzymes such as phospholipase C to produce signaling molecules like inositol triphosphate (IP3) and diacylglycerol (DAG), which are important in intracellular signal transduction [57]. Cholesterol is an important component of cell membranes and also serves as a precursor for steroid hormones. In tumor cells, the metabolism and storage of cholesterol are closely related to cell proliferation and survival [58]. Tumor cells reprogram cholesterol metabolism to meet the demands of their rapid proliferation. This metabolic reprogramming not only supports the biosynthesis of cell membranes but also regulates intracellular signaling by producing bioactive metabolites, such as oxidized cholesterol [59].

In addition, certain fatty acids and their derivatives can be converted into biologically active substances, such as arachidonic acid metabolites (including prostaglandins, thromboxanes, and leukotrienes), which play a role in inflammatory responses and immune regulation [60]. These fatty acids and their derivatives are generated through specific metabolic pathways. For example, arachidonic acid (a polyunsaturated fatty acid) can be metabolized via the cyclooxygenase (COX) and lipoxygenase (LOX) pathways to produce active substances such as prostaglandins, thromboxanes, and leukotrienes.

### 3.5 Impact of Lipid Metabolism on the Tumor Microenvironment (TME)

Lipid metabolism reprogramming also affects the tumor microenvironment (TME), where it interacts with various cellular components to support cancer progression. Cancer‐associated fibroblasts (CAFs), a key component of the TME, secrete fatty acids and regulate lipid metabolism to enhance the growth and invasion of cancer cells [61, 62, 63]. For example, CAFs upregulate the expression of genes related to fatty acid synthesis, providing additional lipids for cancer cell utilization [64, 65].

Immune cells within the TME, such as tumor‐associated macrophages (TAMs), also modulate lipid metabolism through metabolic crosstalk [66]. Upon polarization to the M2 phenotype, TAMs secrete pro‐inflammatory factors like IL‐6, which further promote the growth and migration of cancer cells [25, 67]. This interplay between immune cells and lipid metabolism underscores the complexity of the TME's role in cancer progression [41, 68]. Understanding the genetic and metabolic interactions between cancer cells and immune cells within the TME can reveal new therapeutic targets and strategies [42].

### 3.6 Relationship Between Lipid Metabolism Dysregulation and Cancer Invasion

The remodeling of lipid metabolism plays a central role in cancer invasion and metastasis. Alterations in membrane lipid composition and fluidity enhance the ability of cancer cells to breach the basement membrane barrier [69]. Additionally, lipid‐derived signaling molecules, such as lysophosphatidic acid (LPA), stimulate cytoskeletal reorganization and increase cellular adhesion, facilitating cancer cell migration and metastatic spread [70, 71, 72]. The genetic mutations driving these metabolic alterations can further influence the invasive potential of cancer cells, highlighting the importance of integrating genetic and metabolic analyses in studying cancer metastasis [73].

Moreover, abnormal lipid metabolism is associated with resistance to chemotherapy. Studies have shown that cancer cells with increased lipid storage exhibit greater resistance to chemotherapeutic agents, suggesting that changes in lipid metabolism may represent a key mechanism underlying drug resistance [74, 75, 76]. Targeting lipid metabolic pathways may therefore offer a novel strategy for overcoming treatment resistance and mitigating cancer metastasis.

## 4 Altered Lipid Metabolism in Gastrointestinal Malignancies

Alterations in lipid metabolism in gastrointestinal malignancies mainly include processes such as enhanced de novo lipogenesis, fatty acid oxidation, and cholesterol synthesis and uptake (Table 1). Enhanced de novo lipogenesis is one of the critical mechanisms supporting the rapid proliferation and survival of cancer cells. By increasing lipid synthesis, cancer cells secure the materials required to construct cell membranes and signaling molecules, thereby accelerating their division. Additionally, fatty acids, as a highly efficient energy source, can be oxidized to produce ATP, further supporting the energetic needs of cancer cells.

**TABLE 1:** Main pathways for the dysregulation of lipid metabolism in gastrointestinal cancers.

Metabolic pathway	Alterations in metabolic pathways	Potential impact	Related enzymes/proteins	Reference
FAS	Up‐regulation	Provide cellular membrane components and signaling molecules; support tumor cell proliferation	FASN, ACC	[20, 21, 22, 23, 24]
FAO	Up‐regulation	Affect the modes of energy production, adapt to hypoxic environments	AMPK, CPT1, HIF‐1α	[35, 36, 52]
Cholesterol metabolism	Enhanced synthesis and absorption	Enhanced synthesis and absorption help maintain cellular membrane stability and participate in hormone synthesis	HMGCR, LDLR, SREBP‐2, ACAT1	[77, 78, 79]

Recent studies have demonstrated that FASN and ACC exhibit differential expression patterns across gastrointestinal cancer types [21, 23, 80]. For example, in hepatocellular carcinoma (HCC), FASN expression is often upregulated and associated with poor prognosis. The regulation of FASN by miR‐195 affects HCC progression by downregulating Wnt expression and inhibiting FASN activity, which impairs tumor cell growth and metastasis [81]. In HCC, ACC is another key enzyme in fatty acid metabolism that is frequently overexpressed, driving the synthesis of malonyl‐CoA, a precursor for fatty acid elongation. The inhibition of FASN and ACC has shown promising therapeutic effects in reducing HCC cell growth and sensitizing tumors to chemotherapy, highlighting the potential of targeting fatty acid metabolism in the treatment of HCC [82].

Recent research has highlighted the differences in the expression of these enzymes in different types of gastrointestinal cancers. For instance, FASN is significantly upregulated in gastric cancer and colorectal cancer, contributing to tumor aggressiveness and poor prognosis [83]. In pancreatic cancer, both FASN and ACC are overexpressed, supporting rapid cell division and survival under nutrient‐poor conditions [84]. The highest levels of the lipogenic enzymes ACLY, ACC, and FASN have been detected in HCC characterized by an aggressive phenotype. These enzymes are generally overexpressed in HCC explants in association with an increase of triglycerides, fatty acids, and cholesterol compared to the adjacent non‐tumoral tissue [85].

The expression levels of FASN and ACC are not only associated with tumor aggressiveness and prognosis but may also serve as predictive biomarkers for the efficacy of immunotherapy. For instance, FASN inhibitors have shown promising antitumor activity in clinical trials, particularly when combined with PD‐L1 inhibitors [86]. These findings provide a strong rationale for the development of novel therapeutic strategies targeting lipid metabolism.

In addition to increased lipid synthesis, enhanced fatty acid uptake also supports the lipid metabolic demands of cancer cells. By overexpressing fatty acid transport proteins such as CD36, cancer cells actively absorb free fatty acids (FFAs) from the tumor microenvironment, providing an additional exogenous lipid reservoir [87]. This mechanism further fuels tumor growth and invasion, underscoring the critical role of lipid metabolism reprogramming in gastrointestinal malignancies.

### 4.1 Fatty Acid Oxidation (FAO)

Fatty acid oxidation (FAO) is a critical metabolic adaptation strategy employed by cancer cells to cope with energy stress or changes in the tumor microenvironment. FAO involves breaking down long‐chain fatty acids into acetyl‐CoA, which then enters the tricarboxylic acid (TCA) cycle to generate ATP, providing a sustained energy supply for cancer cells.

Studies have shown that cancer cells can induce lipid reprogramming in surrounding normal cells, increasing fatty acid production to support tumor growth [41]. Tumor‐associated fibroblasts (CAFs) contribute to this process by secreting fatty acids and lipid‐related factors, such as leptin and tumor necrosis factor‐α (TNF‐α), which indirectly enhance cancer cell survival and angiogenesis [88, 89].

Under hypoxic conditions, the expression of hypoxia‐inducible factor‐1α (HIF‐1α) is significantly elevated, upregulating FAO‐related genes to maintain the energy demands of tumor cells [52]. Similarly, during nutrient deprivation, AMP‐activated protein kinase (AMPK) helps cancer cells adapt by suppressing ACC activity and activating the FAO pathway [36]. These regulatory mechanisms enhance FAO, providing metabolic flexibility that enables cancer cells to continue proliferating even under adverse conditions. Gastric cancer cells also exhibit enhanced FAO to cope with energy stress. Studies have shown that hypoxia‐induced HIF‐1α upregulates FAO‐related genes in gastric cancer, providing metabolic flexibility for tumor cells under adverse conditions [90, 91].

### 4.2 Cholesterol Synthesis and Uptake

Cholesterol plays a crucial role as a regulator of cell membrane fluidity and serves as a precursor for the synthesis of steroid hormones and bile acids. In malignancies, abnormalities in cholesterol metabolism manifest as enhanced endogenous cholesterol synthesis and increased exogenous cholesterol uptake [37].

Cholesterol synthesis is regulated by sterol regulatory element‐binding protein‐2 (SREBP‐2), a transcription factor that activates the expression of genes involved in cholesterol biosynthesis. In cancer cells, aberrant activation of SREBP‐2 leads to increased expression of HMG‐CoA reductase (HMGCR), the rate‐limiting enzyme for cholesterol synthesis, thereby promoting cholesterol production [45, 77].

Additionally, the upregulation of low‐density lipoprotein receptors (LDLR) in colorectal cancer enhances the efficiency of cholesterol uptake from the bloodstream [78]. Once internalized, free cholesterol is esterified by acyl‐CoA: cholesterol acyltransferase 1 (ACAT1) and stored as cholesterol esters in lipid droplets. The overexpression of ACAT1 further exacerbates cholesterol accumulation within tumor cells, contributing to tumor progression [79]. In gastric cancer, the upregulation of SREBP‐2 and HMGCR promotes cholesterol synthesis, while increased LDLR expression enhances cholesterol uptake from the bloodstream. These alterations contribute to tumor progression and are associated with poor prognosis [92, 93].

### 4.3 Lipid Signaling and Cancer Progression

Lipids serve not only as structural and energy molecules but also play a pivotal role in regulating signaling pathways that drive malignant behaviors in cancer cells [56]. In gastrointestinal cancers, phosphatidylinositol (PI) and its derivatives are integral to the PI3K/Akt/mTOR signaling pathway. Phosphatidylinositol‐3,4,5‐trisphosphate (PIP3), a key mediator of the PI3K/Akt pathway, activates downstream Akt kinases. This activation promotes cancer cell proliferation, anti‐apoptotic mechanisms, and enhanced migratory capabilities, facilitating tumor growth and progression [54, 55].

Additionally, lysophosphatidic acid (LPA) acts as a potent pro‐angiogenic and pro‐invasive molecule with a critical role in gastrointestinal cancer metastasis. LPA interacts with its receptors, such as LPA1 and LPA3, to activate multiple signaling pathways, thereby promoting cancer cell migration and invasion [94]. In gastric cancer, elevated levels of PIP3 and LPA are linked to increased cell proliferation, migration, and invasion [95]. These lipid mediators serve as potential prognostic biomarkers and therapeutic targets for gastric cancer [96].

#### 4.3.1 Lipid‐Related Biomarkers in Gastrointestinal Cancers

Alterations in lipid metabolism are common metabolic characteristics in gastrointestinal (GI) malignancies, and these changes can be reflected through specific lipid profiles, making them potential diagnostic biomarkers [97, 98, 99] (Table 2).

**TABLE 2:** Lipid biomarkers in gastrointestinal cancers.

Lipid biomarkers	Type	Relationship with gastrointestinal cancers	Diagnosis/prognosis/response to treatment	Reference
FASN	Enzyme	Up‐regulation and involved in tumor cell proliferation, survival, and migration	High expression indicates an increased tumor risk; an indicator of poor prognosis; predicting disease progression or evaluating the effect of neoadjuvant chemotherapy	[100, 101]
PIP3	Enzyme	Activation and promote cell growth and survival	Activating mutations may indicate a specific subtype; the association has a poor prognosis	[102, 103, 104]
TG	Serum lipids	The level may be elevated	Abnormal levels may indicate metabolic disorders; they should be evaluated with other indicators	[105, 106, 107]
FFAs	Serum lipids	Level changes, reflecting the altered metabolism	Level changes can be used as biomarkers; specific trends need further study	[108]
Cholesterol	Serum lipids	The levels may be elevated, involving cell membrane stability	High cholesterol levels may indicate a risk; high levels may be associated with a poor prognosis	[109, 110, 111, 112]
LPA	Lipid media	High expression in tumors, possibly involved in multiple pro‐cancer signaling pathways through regulation of downstream fatty acid network system	May be associated with tumor proliferation, apoptosis and invasion ability; as potential diagnostic and prognostic markers	[71, 72]
PGE2	Lipid media	Plays a role in the pathogenesis of gastrointestinal tumors, and is related to tumor cell proliferation, migration, and invasion	May indicate the risk and prognosis of tumors, with high levels potentially associated with poor prognosis	[113]
SREBP‐1	Nuclear factor	Regulating the biosynthesis of fatty acids, triglycerides, and cholesterol	May be related with proliferation, survival and migration of tumor cells; possibly as an indicator of poor prognosis	[114]

Studies have indicated that low levels of high‐density lipoprotein cholesterol (HDL‐C) may be associated with a higher risk of GI cancers [109, 110, 115]. For example, a study by Zhang et al. [111] found a significant correlation between low HDL‐C levels and an increased risk of colorectal cancer. In contrast, high levels of low‐density lipoprotein cholesterol (LDL‐C) are thought to promote cancer development [112], although evidence for LDL‐C's role is inconsistent, and further research is needed to clarify its exact contribution [7].

Additionally, elevated triglyceride (TG) levels are closely linked to insulin resistance, obesity, and metabolic syndrome—key risk factors for GI cancers [105, 106]. Studies have shown that individuals with hypertriglyceridemia have a significantly higher risk of developing colorectal cancer [107]. These lipid profile alterations hold promise for providing precise tools for the early detection of GI cancers and may become important diagnostic methods in personalized medicine.

#### 4.3.2 Lipid Metabolic Enzymes and Metabolite Biomarkers

Fatty acid synthase (FASN): As previously discussed, FASN is frequently overexpressed in GI cancers and plays a crucial role in metabolic reprogramming [116, 117]. Clinically, it has emerged as a promising biomarker for predicting tumor aggressiveness, response to neoadjuvant chemotherapy, and overall prognosis.

Sterol regulatory element‐binding protein 1 (SREBP‐1): SREBP‐1 is a critical transcription factor that controls the expression of genes involved in cholesterol and fatty acid synthesis. In colorectal cancer, increased SREBP‐1 activity promotes lipid accumulation within tumor cells and reduces their sensitivity to therapeutic agents [114]. Consequently, the aberrant activation of SREBP‐1 serves as an important biomarker for detecting early‐stage lesions and potentially guiding therapeutic decisions.

Free fatty acids (FFAs) and sphingolipids: Elevated levels of FFAs are often associated with lipid metabolism dysregulation within the tumor microenvironment. FFAs not only provide energy for tumor cells but also participate in signaling pathways that promote cancer cell proliferation and migration [108]. Additionally, sphingolipid metabolites such as ceramides play dual regulatory roles in apoptosis and proliferation [118]. Investigating these metabolites can aid in identifying novel therapeutic targets for GI cancers.

#### 4.3.3 Lipid‐Based Prognostic Biomarkers

Phosphatidylinositol‐3,4,5‐trisphosphate (PIP3): The PI3K/Akt/mTOR signaling pathway plays a central role in cancer cell growth, survival, and metabolism [102, 103, 119, 120]. As a key mediator of this pathway, elevated levels of PIP3 are strongly associated with poor prognosis in gastrointestinal (GI) cancers [104]. Dynamic monitoring of PIP3 levels can help assess treatment response and predict the risk of recurrence, providing valuable insights for patient management.

Lysophosphatidic acid (LPA): LPA regulates cancer cell migration, invasion, and angiogenesis by activating specific receptors, such as LPA1 and LPA3. Increased LPA concentrations are linked to the high invasiveness and metastatic potential of GI cancer cells [71, 72]. As a result, LPA serves as an important prognostic indicator for evaluating tumor progression and aggressiveness.

Prostaglandin E2 (PGE2): PGE2, a lipid signaling molecule derived from arachidonic acid metabolism, is commonly elevated in GI cancers. High levels of PGE2 promote tumor growth, angiogenesis, and immune evasion and are significantly associated with poor prognosis [113]. Monitoring PGE2 levels may provide critical insights into tumor progression and guide therapeutic strategies.

## 5 The Interplay Between Lipid Metabolism, Tumor Microenvironment, and Lifestyle Factors in Gastrointestinal Cancers

### 5.1 Lipid Metabolism and TME Interactions

**FIGURE 2:**
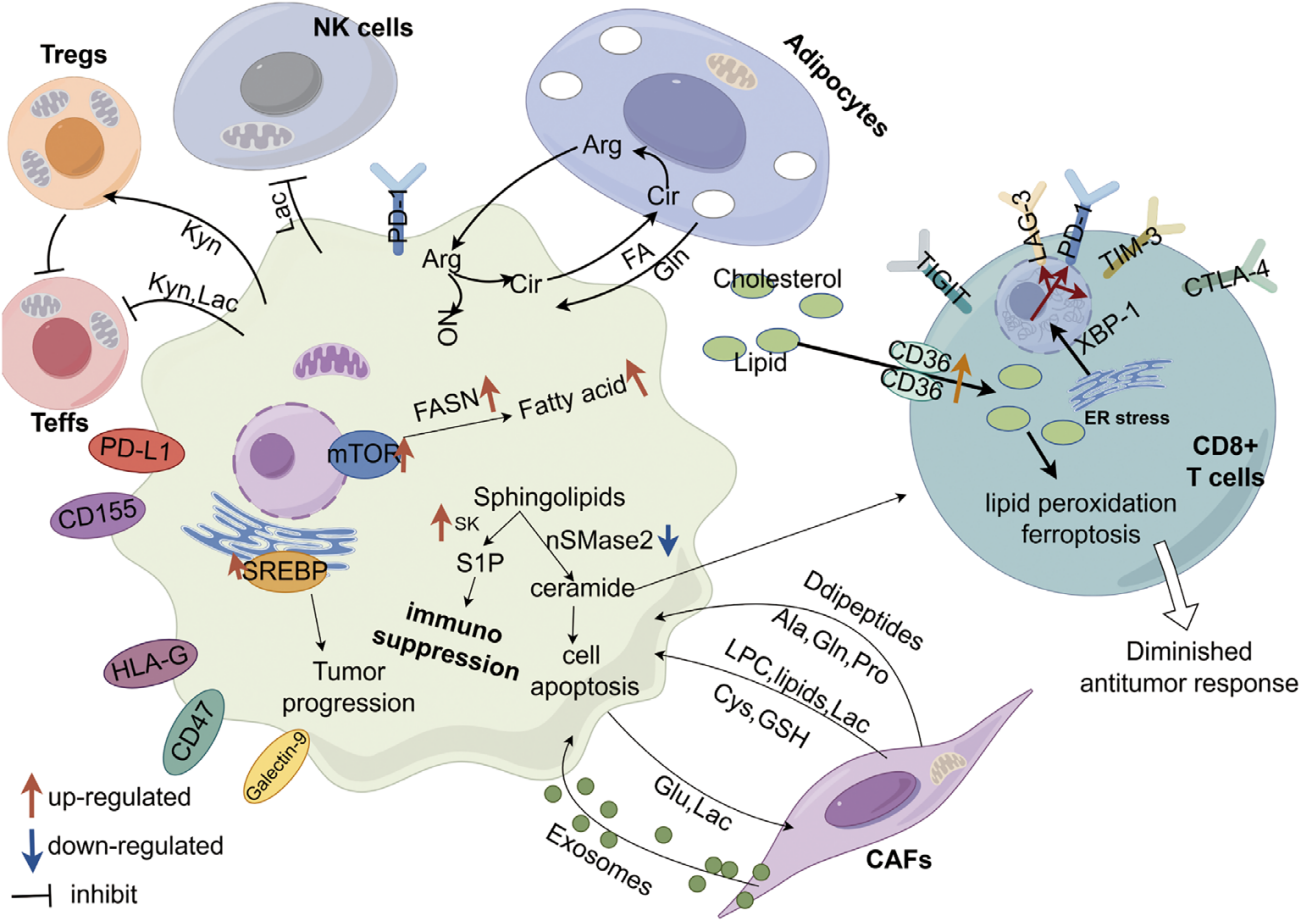
The interaction network between tumor microenvironment (TME) and lipid metabolism.

Lipid metabolism and the tumor microenvironment (TME) interact in a complex, bidirectional manner, profoundly influencing the development and progression of gastrointestinal malignancies (GI cancers; Figure 2). The TME supports cancer cells through metabolic exchanges while promoting tumor growth via mechanisms such as inflammation, immune evasion, and angiogenesis. Stromal and immune cells within the TME, such as cancer‐associated fibroblasts (CAFs) and tumor‐associated macrophages (TAMs), secrete lipids and related metabolites that enhance cancer cell proliferation, invasion, and metastasis [25]. At the same time, cancer cells reshape the TME by altering lipid metabolism, creating an environment that favors their survival and spread [41].

### 5.2 Lipid Metabolism and Immune Cell Function

Emerging evidence suggests that lipid metabolism within the tumor microenvironment (TME) not only influences stromal cells such as cancer‐associated fibroblasts (CAFs) and tumor‐associated macrophages (TAMs), but also plays a pivotal role in modulating immune cell activity. Aberrant lipid availability and accumulation can affect immune cell metabolism, differentiation, and effector functions, thereby shaping the immunosuppressive landscape of gastrointestinal cancers [121].

#### 5.2.1 Effect of Lipid Metabolism on Immune Cells

Lipid metabolic reprogramming in the TME directly impacts the antitumor activity of T cells and NK cells. High levels of lipid metabolites, particularly free fatty acids and oxidized lipids, can impair CD8^+^ cytotoxic T lymphocyte (CTL) function by inducing mitochondrial dysfunction, lipid peroxidation, and oxidative stress [122]. For example, elevated lipid accumulation in CD8^+^ T cells within the TME impairs their cytotoxic function and promotes exhaustion, thereby diminishing the efficacy of immunotherapy [123]. This leads to T cell exhaustion and diminished capacity to eliminate tumor cells. Similarly, elevated lipid uptake by regulatory T cells (Tregs) enhances their immunosuppressive activity, further dampening the antitumor immune response.

In NK cells, dysregulated lipid metabolism has been associated with reduced cytotoxicity and impaired interferon‐γ (IFN‐γ) production, weakening their role in tumor surveillance. These findings suggest that targeting lipid metabolic pathways may improve the efficacy of immunotherapies, including immune checkpoint inhibitors.

#### 5.2.2 Lipid Metabolism in Immune Evasion and Pro‐Angiogenic Signaling

Lipid‐derived signaling molecules also contribute to immune escape and angiogenesis within the TME. Prostaglandin E2 (PGE2), a product of arachidonic acid metabolism, promotes immunosuppression by inhibiting the maturation and function of dendritic cells and reducing T cell activation. Additionally, PGE2 facilitates neovascularization by promoting endothelial cell proliferation through AMPK‐dependent pathways [124].

Lysophosphatidic acid (LPA), another bioactive lipid, has been shown to promote tumor angiogenesis via activation of YAP/TAZ signaling, and to modulate immune responses by influencing cytokine secretion and lymphocyte migration [125]. These dual roles highlight the importance of lipid signaling networks in establishing a tumor‐promoting microenvironment. Therapeutic targeting of these lipid mediators may offer synergistic benefits when combined with immunomodulatory or anti‐angiogenic treatments.

### 5.3 Lipid Metabolism and ECM Remodeling

Within the TME, CAFs exhibit heightened lipid metabolic activity, secreting fatty acids and lipid factors like leptin to provide additional energy for rapidly proliferating cancer cells [61]. They also remodel the extracellular matrix (ECM) to support cancer cell migration and invasion [62, 100]. Similarly, TAMs in their M2‐polarized state secrete pro‐inflammatory cytokines such as IL‐10 and angiogenic factors like VEGF, facilitating immune evasion and angiogenesis. TAMs also accumulate lipid metabolites, such as free fatty acids and cholesterol, which further sustain cancer cell growth. Lipid metabolites, including prostaglandin E2 (PGE2) and lysophosphatidic acid (LPA), enhance endothelial cell proliferation and migration, promoting angiogenesis [101]. The resulting new blood vessels supply nutrients to cancer cells and facilitate their spread to distant sites [25].

Cancer cells actively remodel TME functions by secreting lipid‐related signaling molecules [63]. Inflammatory cytokines like IL‐6 and TNF‐α released by cancer cells activate adipocytes in the TME, shifting them from storage to lipolysis mode and releasing free fatty acids for cancer cells to utilize [56]. Cancer cells also secrete transforming growth factor β (TGF‐β) and chemokines such as CCL2 and CXCL8, which induce fibroblasts to transition into CAFs and macrophages into TAMs. These reprogrammed cells enhance exogenous lipid uptake and pass these resources to cancer cells [62]. Lipid metabolites, such as phospholipids and fatty acid derivatives, further modify the ECM, increasing its stiffness and allowing cancer cells to breach the basement membrane, enhancing their invasiveness and migratory capacity.

### 5.4 Cancer Cells' Influence on the TME

Cancer cells actively remodel TME functions by secreting lipid‐related signaling molecules [63]. Inflammatory cytokines like IL‐6 and TNF‐α released by cancer cells activate adipocytes in the TME, shifting them from storage to lipolysis mode and releasing free fatty acids for cancer cells to utilize [56]. Cancer cells also secrete transforming growth factor β (TGF‐β) and chemokines such as CCL2 and CXCL8, which induce fibroblasts to transition into CAFs and macrophages into TAMs. These reprogrammed cells enhance exogenous lipid uptake and pass these resources to cancer cells [62].

### 5.5 Environmental and Lifestyle Factors

Environmental and lifestyle factors also play a role in shaping lipid metabolism and GI cancer risk. Alcohol consumption is a significant risk factor, indirectly increasing GI cancer risk through disruptions in hepatic lipid metabolism [4]. Evidence suggests that higher alcohol intake correlates with greater tumor invasiveness and metastatic potential [126]. Obesity, which has become a major determinant of certain cancers, significantly increases GI cancer risk, particularly colorectal cancer, through insulin resistance and lipid metabolism dysregulation [127, 128, 129, 130]. Obese individuals often experience shorter overall survival compared to those with normal weight [131]. Intentional weight loss may help reduce cancer incidence and mortality, particularly in women [132].

## 6 Therapeutic Strategies Targeting Lipid Metabolism in Gastrointestinal Cancers

### 6.1 Targeting Lipid Metabolism in GI Cancer Treatment

Pharmacological inhibitors targeting lipid metabolism offer a promising avenue for the treatment of gastrointestinal malignancies (GI cancers). These inhibitors disrupt tumor cell energy supply and biosynthetic pathways by blocking fatty acid synthesis, oxidation, or cholesterol metabolism, demonstrating significant antitumor potential.

**FIGURE 3:**
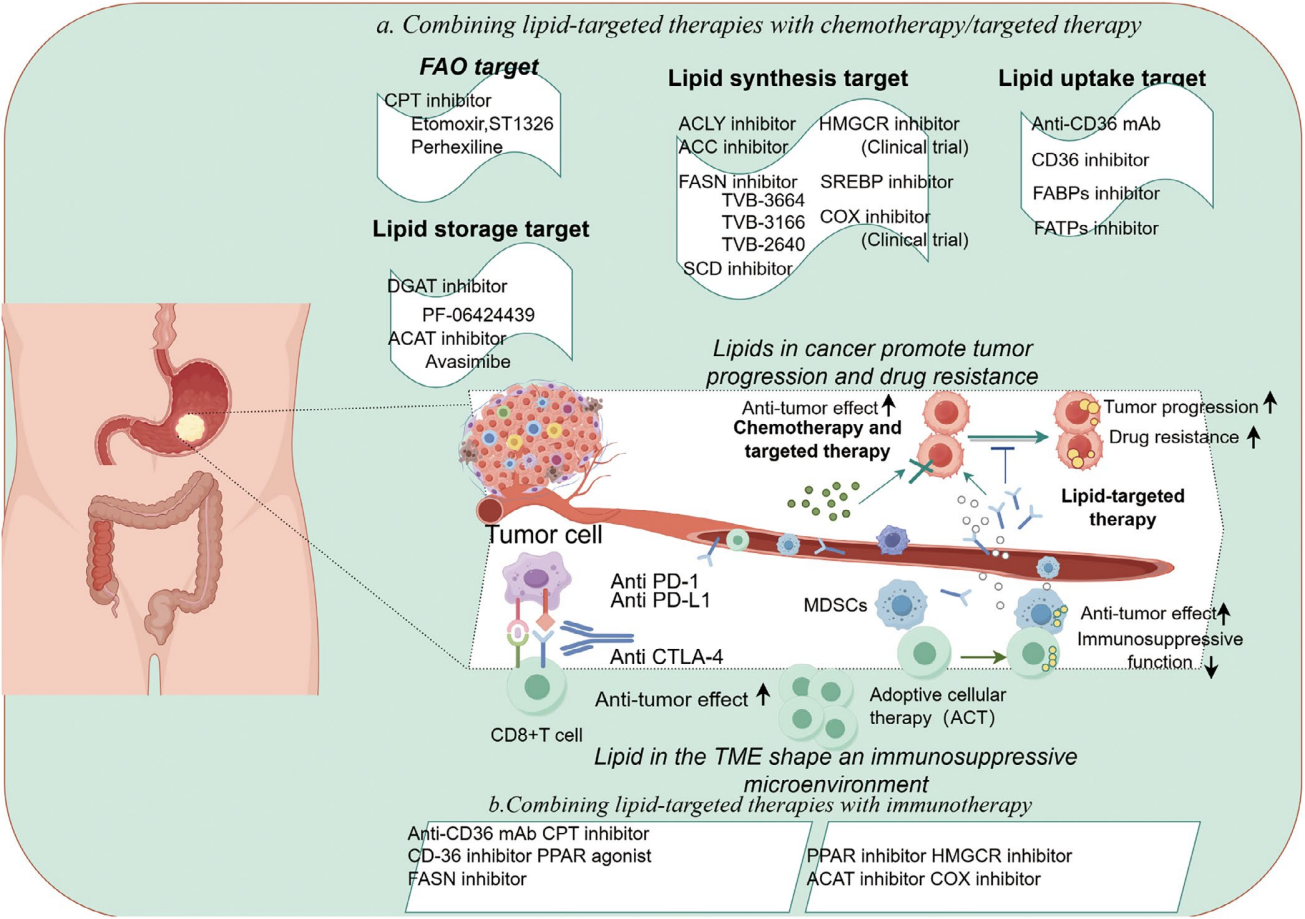
Lipid‐targeted therapy in combination with chemotherapy, targeted therapy, and immunotherapy. Recent studies demonstrate that inhibitors of lipid metabolism enhance the efficacy of conventional anti‐tumor therapies, including chemotherapy, molecular targeted agents, and immunotherapy. Key targets include lipid uptake (CD36, FABPs, and FATPs), lipid synthesis (ACLY, ACC, FASN, SCD, SREBP, HMGCR, and COX), fatty acid oxidation (FAO and CPT1), and lipid storage in lipid droplets (DGAT and ACAT). These strategies offer promising avenues to overcome drug resistance, reshape the tumor microenvironment, and improve therapeutic outcomes in gastrointestinal cancers.

Recent studies have highlighted the potential of targeting lipid metabolism in cancer treatment. Advances in lipidomics and lipid biology have revealed novel pathways and molecules that can be exploited for therapeutic purposes [133]. For example, inhibitors of lipid synthesis or metabolism have shown promise in preclinical studies and are currently being investigated in clinical trials. For instance, fatty acid synthase (FASN) inhibitors like TVB‐2640, currently in clinical trials, show promise in reducing tumor growth and enhancing chemotherapy efficacy [134]. Similarly, acetyl‐CoA carboxylase (ACC) inhibitors, such as CP‐610431, exhibit potent antitumor activity in preclinical studies and may be even more effective when combined with immunotherapy or chemotherapy, especially in tumors with highly active lipid metabolism [135]. Carnitine palmitoyltransferase 1A (CPT1A) inhibitors, like ST1326, have demonstrated early clinical success by weakening tumor cell survival under hypoxic and nutrient‐deprived conditions through fatty acid oxidation (FAO) inhibition (Figure 3) [136].

### 6.2 The Role of Dietary Lipids in Cancer

Additionally, dietary lipids play a significant role in cancer development and progression. Certain types of fats, such as saturated fats (SFAs) and trans fats, have been linked to an increased risk of various cancers, including those of the gastrointestinal tract [137]. On the other hand, unsaturated fats, particularly omega‐3 fatty acids, have been shown to have protective effects [138]. A recent systematic review and meta‐analysis revealed a significant positive correlation between elevated levels of total SFAs and cancer risk (OR of 1.294; 95% CI: 1.182–1.416; *p*‐value less than 0.001) [139]. Elevated levels of specific SFA subtypes, such as C14:0, C16:0, and C18:0, were also implicated in increased cancer risk. This association was particularly strong for breast, prostate, and colorectal cancers. The overconsumption of dietary SFAs can lead to obesity, which in turn reduces the number and anti‐tumor activity of CD8+ T cells within tumors, accelerating tumor growth [140].

In contrast, certain bioactive compounds also hold therapeutic potential. Omega‐3 polyunsaturated fatty acids (ω‐3 PUFAs), known for their anti‐inflammatory properties, can induce colorectal cancer cell apoptosis by increasing reactive oxygen species (ROS) and reducing chronic inflammation that drives tumor progression [138, 141]. Esculin, a natural compound derived from the bark of the ash tree, has shown significant antitumor effects in colorectal cancer models by inducing apoptosis and ferroptosis [142]. Statins may have anti‐cancer effects by blocking the mevalonate pathway and reducing tumor cell growth [143, 144].

### 6.3 Combination Therapy Strategy

Combining lipid metabolism‐targeted therapy with conventional treatments such as chemotherapy and immunotherapy holds great promise. Targeting lipid metabolism can diminish cancer cells' metabolic flexibility, enhancing the cytotoxic effects of chemotherapeutic drugs and reversing chemotherapy resistance [145, 146]. Additionally, lipid metabolism influences immune cell behavior, offering new synergies with immunotherapy [147]. For example, combining lipid‐targeting drugs with immune checkpoint inhibitors, such as PD‐1/PD‐L1 blockers, has shown potential in enhancing antitumor immune responses. Personalized therapeutic strategies tailored to individual genetic backgrounds and lipid metabolic profiles are also essential. For instance, certain single nucleotide polymorphisms (SNPs) can affect metabolic enzyme activity, influencing a patient's response to specific therapies [148, 149]. Leveraging lipidomic profiles, genetic data, and clinical histories may enable the development of more effective and less toxic individualized treatments.

### 6.4 Challenges and Future Directions

Despite these advances, challenges remain. Many lipid metabolic enzymes are expressed in normal tissues as well as cancer cells, raising concerns about off‐target effects and toxicity [150]. Over‐inhibition of lipid metabolism could disrupt systemic energy balance, impair immune function, and increase the risk of infections or complications. Furthermore, comorbidities such as metabolic syndrome and diabetes, commonly observed in GI cancer patients, exacerbate lipid metabolism dysregulation and complicate treatment [151, 152].

Meanwhile, the heterogeneity of tumor cells poses a significant challenge in studying lipid metabolism in cancer. Tumor cells within a single tumor can exhibit diverse metabolic profiles, influenced by genetic mutations, epigenetic modifications, and microenvironmental factors. This heterogeneity can lead to differential responses to lipid metabolism‐targeting therapies, complicating the development of effective treatment strategies [153].

Moreover, lipid metabolism is also intricately linked to other metabolic pathways and cellular processes. Lipids interact with proteins, carbohydrates, and nucleic acids, forming complex networks that regulate cellular function. For example, lipid metabolites such as prostaglandin E2 (PGE2) and lysophosphatidic acid (LPA) can modulate immune cell function and promote angiogenesis, highlighting the need for a comprehensive understanding of these interactions [121]. Additionally, lipid metabolism can influence epigenetic modifications through fatty acylation of key molecules, further complicating the study of cancer metabolism.

Future research directions should focus on standardizing lipidomic analysis techniques to improve diagnostic and therapeutic monitoring accuracy. Optimizing combination therapies, such as integrating lipid‐targeting agents with radiotherapy or chemotherapy, could unlock synergistic effects. Meanwhile, addressing the challenges of tumor cell heterogeneity and complex lipid interactions will be crucial for advancing our understanding of lipid metabolism in cancer and developing more effective treatments [153]. Additionally, further investigation into the dynamics of lipid metabolism within the tumor microenvironment (TME) may reveal new therapeutic targets and improve outcomes.

## 7 Conclusions

This review systematically highlights the multifaceted role of lipid metabolism in gastrointestinal malignancies (GI cancers), underscoring its immense potential as a disease driver, diagnostic tool, and therapeutic target. Lipid metabolism supports cancer growth and shapes the TME, influencing tumor progression and therapy response. Various lipid metabolism‐related bioactive compounds and targeted therapies have demonstrated promising antitumor activity, offering novel approaches for the clinical management of GI cancers.

The clinical application of lipid‐targeted therapies and lipid‐based biomarkers is gradually transforming the diagnostic and therapeutic landscape for GI cancers. Targeting lipid metabolism can disrupt tumor energy supply and signaling networks to enhance therapeutic efficacy, while lipid‐related biomarkers enable early detection, prognosis evaluation, and real‐time treatment monitoring, driving the advancement of personalized medicine. However, these strategies face challenges, including off‐target effects, genetic heterogeneity, and the impact of comorbid chronic conditions on therapeutic outcomes.

Future research should focus on key areas to overcome these hurdles and unlock the full potential of lipid metabolism in cancer management. First, detailed mechanistic studies are needed to elucidate the molecular pathways by which lipid metabolism contributes to tumorigenesis, TME remodeling, immune interactions, angiogenesis, and drug resistance. Second, developing more efficient and safer lipid metabolism‐targeted drugs, particularly those with precise inhibition of specific enzymes or pathways, will be critical, along with validating their use in combination therapies. Third, standardizing the use of lipid biomarkers through optimized lipidomic analysis techniques and streamlined diagnostic workflows will enable more accurate diagnosis and treatment monitoring. Lastly, integrating lipid profiles, genetic data, and TME characteristics will allow the design of personalized therapeutic strategies, maximizing efficacy while minimizing adverse effects.

A deeper understanding of lipid metabolism can lead to new therapeutic targets and better GI cancer outcomes. The research on lipid metabolism also provides valuable guidance for managing other cancer types. Moving forward, multidisciplinary collaboration between basic science and clinical practice will be essential to drive breakthroughs in this rapidly evolving field.

## Author Contributions

**Yan An:** conceptualization (equal), data curation (equal), methodology (equal), writing – original draft (equal). **Huihui Song:** data curation (equal), investigation (equal). **Hongyan Qiu:** data curation (equal), investigation (equal). **Jun Jiang:** supervision (equal), writing – review and editing (equal). **Junfeng Shi:** supervision (equal), writing – review and editing (equal).

## Disclosure

Disclaimer/Publisher's Note: The statements, opinions, and data contained in all publications are solely those of the individual author(s) and contributor(s) and not of MDPI and/or the editor(s). MDPI and/or the editor(s) disclaim responsibility for any injury to people or property resulting from any ideas, methods, instructions, or products referred to in the content.

## Ethics Statement

The authors have nothing to report.

## Consent

The authors have nothing to report.

## Conflicts of Interest

The authors declare no conflicts of interest.

## Data Availability

The authors have nothing to report.
